# Investigating Lushan Earthquake Victims’ Individual Behavior Response and Rescue Organization

**DOI:** 10.3390/ijerph14121556

**Published:** 2017-12-11

**Authors:** Peng Kang, Yipeng Lv, Qiangyu Deng, Yuan Liu, Yi Zhang, Xu Liu, Lulu Zhang

**Affiliations:** Department of Military Health Service Management, College of Military Health Service Management, Second Military Medical University, Shanghai 200433, China; kpkp315@163.com (P.K.); epengl@163.com (Y.L.); smmudqy@163.com (Q.D.); yawnlau@126.com (Y.L.); 18602160005@126.com (Y.Z.); aqualau@126.com (X.L.)

**Keywords:** earthquake victims, individual behavior, injury, rescue efforts

## Abstract

Research concerning the impact of earthquake victims’ individual behavior and its association with earthquake-related injuries is lacking. This study examined this relationship along with effectiveness of earthquake rescue measures. The six most severely destroyed townships during the Lushan earthquake were examined; 28 villages and three earthquake victims’ settlement camp areas were selected as research areas. Inclusion criteria comprised living in Lushan county for a longtime, living in Lushan county during the 2013 Lushan earthquake, and having one’s home destroyed. Earthquake victims with an intellectual disability or communication problems were excluded. The earthquake victims (N (number) = 5165, male = 2396) completed a questionnaire (response rate: 94.7%). Among them, 209 were injured (5.61%). Teachers (*p* < 0.0001, OR (odds ratios) = 3.33) and medical staff (*p* = 0.001, OR = 4.35) were more vulnerable to the earthquake than were farmers. Individual behavior was directly related to injuries, such as the first reaction after earthquake and fear. There is an obvious connection between earthquake-related injury and individual behavior characteristics. It is strongly suggested that victims receive mental health support from medical practitioners and the government to minimize negative effects. The initial reaction after an earthquake also played a vital role in victims’ trauma; therefore, earthquake-related experience and education may prevent injuries. Self-aid and mutual help played key roles in emergency, medical rescue efforts.

## 1. Introduction

Natural disasters, especially earthquakes, cause vast destruction, often resulting in massive casualties and economic loss. Researchers pay great attention to injuries and rescue efforts after earthquakes [1,2,3,4]; however, most scholars have focused on analyzing the earthquake trauma structure and its influencing factors, including the objective mechanisms after buildings collapse and the structure and timing of disaster relief deployment [5,6]. Consequently, the data collected by medical institutions are the main source of research data. However, hospitals in earthquake areas, especially first-level medical institutions, are in a chaotic state after an earthquake [1,4], which leads to data collection errors and omissions. To obtain data about earthquake trauma, many researchers can only conduct simple, cross-sectional, and epidemiological investigations to determine the objective influencing factors of earthquake trauma [7,8,9]. As for earthquake victims, the subjective influencing factors of trauma and individual behavioral response have also been largely ignored.

In past research, post-earthquake injury evaluation and rescue management were more popular topics than proactive plans aimed at protecting individuals [10,11,12]. However, as we know, pre-disaster management is always much better and efficient than post-disaster rescue for saving both people’s lives and property. Thus, identifying the link between individual response and injury and intervening before the disaster can help to reduce the injury or even prevent deaths. 

In particular, after an earthquake, people tend to be anxious, frightened and overwhelmed [13,14,15]. Such types of behavioral responses may make them more vulnerable to injury. However, current research concerning victims’ behavior is based on hospital interviews or case reports. There is no systemic research with a large sample concentrating on earthquake victims’ individual behavior. Precise and timely deployment of rescue forces to the most severely affected areas is commonly addressed; however, the impact of individual behavior on emergency rescue has been neglected, especially self- and mutual-aid efforts directly after the earthquake. Furthermore, people who live in rural counties in China are not educated regarding well-being, especially how to help themselves and others after an earthquake [11,16]. To what extent self-aid and mutual rescue efforts in rural disaster-affected areas can influence the effect of emergency medical rescue is worth studying.

Consequently, determining the relationships among individual behaviors, like the relationships among victims’ emotions after earthquake, first reaction during the earthquake, earthquake-related experience, self- and mutual-aid, and their injury are the main purpose of our research. We also tried to give some policy suggestions based on our results to the rescue organization after earthquake. This research focuses on the areas in western China most affected by earthquakes. Specifically, we investigated the aftermath of the Lushan earthquake. The six most severe earthquake-affected townships including 26 villages were surveyed with an on-site investigation and a large amount of injury- and rescue-related first-hand data were collected. We attempted to elucidate the relationships among personal characteristics, injury and rescue effectiveness.

## 2. Methods

### 2.1. Study Design

The area most severely affected by earthquake was Lushan county. The six most destroyed townships were selected, including Taiping, Shuangshi, Luyang, Baosheng, Longmen and Qingren. Twenty-eight villages and 3 earthquake victims’ settlement camp areas were selected as the research areas. As family members shared similar experiences as they stayed together at home, we preferred to investigate individuals from different families to get a holistic view of the earthquake and the related injury, which is more efficient. Two-step sampling was used in the research to avoid sampling bias. We used random sampling method both in the first step (choosing the families) and the second step (choosing the specific family member). With the help of Lushan county’s government, we conducted random sampling through the census registration system. We randomly chose 5452 families, which accounted for 33% of all families in Lushan county. Then, the family member whose birthday was closest to the investigation day was chosen as the participant. If the selected participant did not wish to participate for some reason, the individual with the second closest birthday participated, and so on. This sampling method was used until we found a participant from each family. Inclusion criteria comprised living in Lushan county for a long time, living in Lushan county during the 2013 Lushan earthquake, and having one’s home destroyed. Earthquake victims with an intellectual disability or communication problems were excluded.

### 2.2. Data Collection

Three months after the Lushan earthquake in July 2013, our research group arrived at Lushan, Sichuan Province to conduct this research. Since researchers and native residents spoke different dialects, nine graduate students from local universities who had bachelors’ degrees in social science completed a three-day training course so that they would be able to administer the questionnaires competently. The training included the research purpose, explaining consent, instrument design, and communication skills. All students passed the test and conducted the investigation independently.

After participants provided written consent to participate, they completed the questionnaire individually. Researchers explained any confusing questions or recorded the answers if participants could not read. In total, 5165 participants completed the questionnaire (response rate: 94.7%). Ethical approval was granted by the Ethics Committee of Second Military Medical University. The consent procedure was also approved by this committee.

### 2.3. Instruments

The questionnaire used in this study was designed based on the “earthquake survivor questionnaire,” created at the University of Cambridge, the UK, and Islamia College, Pakistan. This questionnaire assesses information about earthquake injury and influencing factors during and after an earthquake. Based on the geographic and cultural characteristics of western China, some questionnaire items were amended and combined with earthquake emergency medical rescue information such as timely medical treatment, medical evacuation, and rescue efficiency and effect. After consulting experts in the emergency rescue field several times, the final edition of the “Lushan earthquake victims” questionnaire was created. Although the questionnaire comprised six aspects, only three were included in this research: (1) demographic information such as name, sex, education, occupation, marriage, and so on; (2) personal behavioral characteristics such as experience with the 2008 Wenchuan earthquake, earthquake injury, earthquake evacuation training, fear level, first reaction, whether they were trapped, and their family members’ situation; and (3) victims’ injury and treatment including injury time, on-site medical treatment, first evacuation experience, and so on.

### 2.4. Data Analysis

All data were analyzed via SAS version 9.0 (SAS Institute Inc., Cary, NC, USA). We first calculated the descriptive statistics (frequencies, percentages, means and standard deviations) to show the basic information of the participants in the investigation. χ² analysis was used in analysis of all multi-category variables. The statistical method of an M:N pair design multivariate logistic regression was used in the multivariate analysis to determine the key factors influencing earthquake injuries. When matched with geographic location (the earthquake-affected area), the analysis can exclude the bias of influence of people’s traits in various geographic locations. Odds ratios (OR) and 95% confidence intervals (CI) were used to evaluate the risk of variables. The criterion for statistical significance was set at *p* = 0.05.

### 2.5. Ethical Statement

All subjects gave their informed consent before they participated in the study. The study was conducted in accordance with the Declaration of Helsinki, and the protocol was approved by the Ethics Committee of Second Military Medical University and the ethical approval code was 2014LL015.

## 3. Results

Participants’ demographic details in terms of their injuries are shown in Table 1. Individuals older than 65 years were significantly more likely to be injured. Teachers and medical staff were more vulnerable to the earthquake than were farmers. 

**Figure 1:**
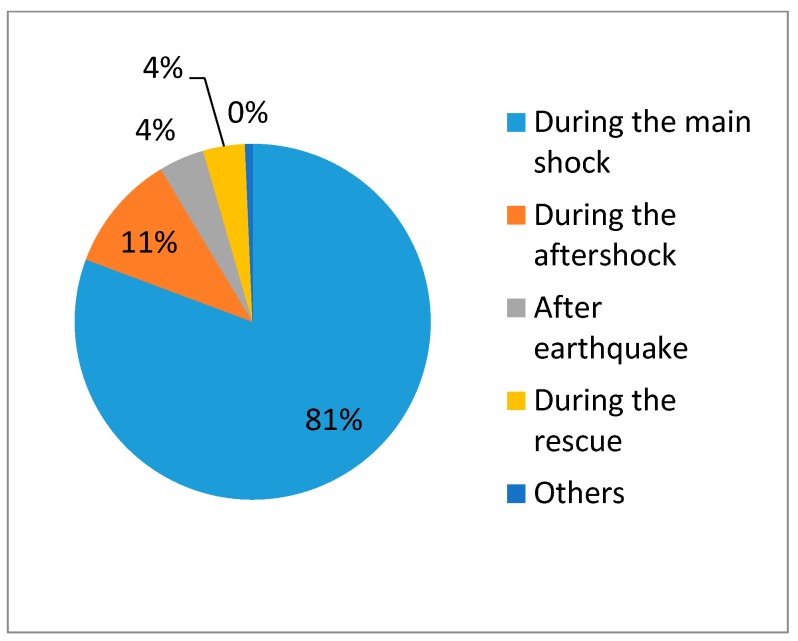
The time at which victims got injured.

Details about participants’ individual characteristics are shown in Table 2. Earthquake-related mental health education was lacking among the victims: only 36.73% of them received pre- and post-earthquake mental health counseling or training. Most victims were terrified during the earthquake. There were wide variations regarding victims’ first reactions after the earthquake occurred. The odds of being injured increased when victims’ fear reached the two highest levels. People were most likely to be injured if their first reaction was to stand up or run out when trapped. Figure 1 shows the time at which victims got injured. Of them, 81% were injured during the earthquake, which has an important relationship with their first reaction. The injury risk rate increased for those who did not experience the 2008 Wenchuan earthquake, indicating that a prior experience of earthquake may protect victims. However, previous injury increased the percentage of those who were injured in the Lushan earthquake by 66%. Furthermore, previously receiving earthquake evacuation training may significantly decrease the risk rate for injury. However, there was no significant difference between injured victims who received mental health education and those who did not.

**Figure 2:**
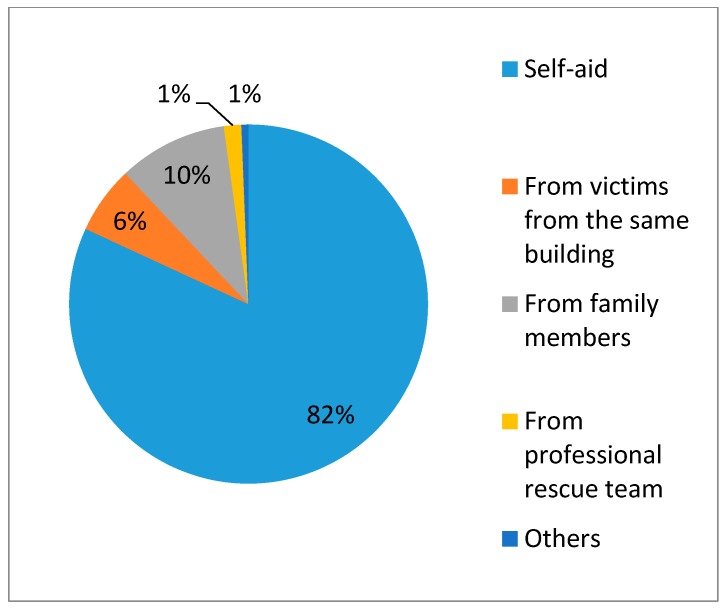
Ways of getting out of the collapsed building.

**Figure 3:**
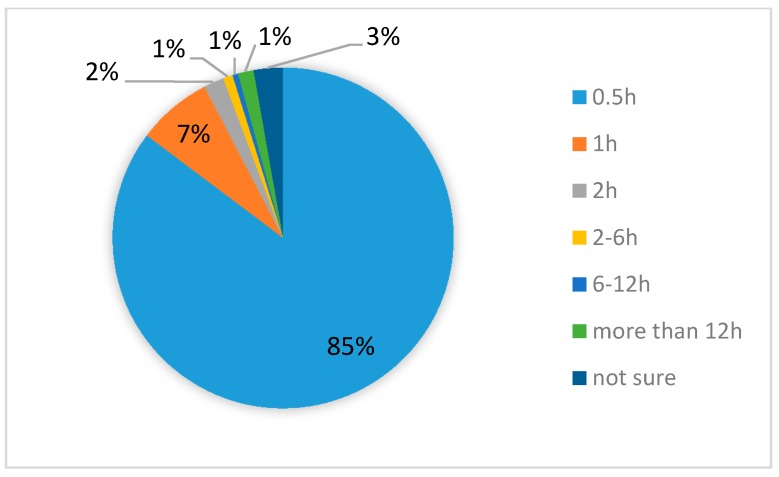
Time taken to get out of the collapsed building (hours).

**Figure 4:**
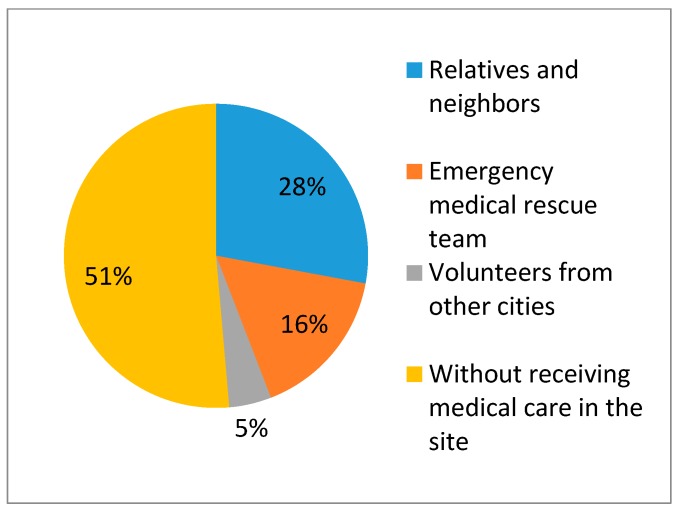
Source of medical care in the site.

Based on the findings of this research, self- and mutual-aid played a critical role. Figure 2 shows how trapped victims were rescued, suggesting that knowing how to help oneself and others is key to saving lives. Furthermore, based on our investigation of rescue information, 85.26% of trapped victims were saved within 30 min while 95% of victims were saved in less than 2 h (Figure 3). Moreover, 78.03% of victims participated in the rescue action immediately after they were saved. Figure 4 shows the result of emergency medical care for the victims in the site. Half of them did not receive emergency medical care on site from the professional medical personnel because the local medical institutions were destroyed. Victims had to rely on themselves for obtaining medical care. 

Among all interviewed victims, 290 were injured during the earthquake, which accounts for 10.45% of the families in the disaster area. The percentage of families that lost a member to death by the earthquake was 1.99%. Importantly, 33.98% of the families ascribed their relatives’ death to their severe injury while more than half of them blamed it on rescue delay due to a traffic jam or a lack of medical resources. Figure 3 and Figure 4 display information about the timing of victims’ injuries and who they received care from, respectively.

## 4. Discussion

Only two demographic factors in the research show an association with injury. It is easy to accept the fact that older people seem more likely to be injured during an earthquake. It is interesting that teachers and medical staff are more likely to be injured. Based on news reports after earthquakes, it can be observed that both these groups are more likely to help the others. The teachers are always responsible for their students’ safety and the medical staff are more likely to stay in the severely destroyed areas trying to help others, which greatly increases their possibility for injury.

Our results revealed that there is an obvious connection between earthquake-related injury and individual behavioral characteristics. In the research, this kind of relationship is mainly reflected in four aspects: the relationships among victims’ emotions after earthquake, first reaction during the earthquake, earthquake-related experience, self- and mutual-aid, and their injury. 

In the emotion part, most victims felt extremely terrified during the earthquake, which added to the risk of injury. Extreme fear may negatively affect people’s cognition and judgment during an earthquake. Further research showed that these victims are also more susceptible to post traumatic stress disorder, which leads to an inferior quality of life [14,17,18]. Consequently, victims require mental health support to minimize these negative effects. Here, by mental health support, we refer to all the methods to help earthquake victims in dealing with their mental problems including depression or severe mental problems to get relief from and cure for their mental problems. Professional mental health support for the victims who experienced an earthquake for the first time can help them to develop a healthier emotional functioning and to start a new life instead of being trapped in the state of sadness; for the victims who had previously suffered an earthquake, mental health can help them to get rid of its terrible memory and develop a better reaction to face the earthquake and their loss.

The first reaction after an earthquake plays a vital role in victims’ experience of trauma. As shown in the results, most of the victims got injured during the main earthquake. Standing up immediately or running around without a destination will increase the risk rate for injury. It is commonly accepted that victims should hide under solid material to avoid being hit by falling objects (e.g., hiding under a desk or solid furniture and protecting one’s head and neck). People should not run around until they are sure that the earthquake has stopped, and the building is safe. Research about the California earthquake in 1979 revealed that 50% of the victims got hurt by hitting desks or doors when running out of the house in a panic. Mahue-Giangreco [19] found that some victims valued their property too much, which led to their injuries. We suggest that people should seek to protect themselves and their families instead of their property. Research about an Armenian earthquake found that successfully running out of an effected area is a protective factor for victims—that people should exit buildings with a plan during an earthquake [2]. The results revealed that individuals’ behavior will be greatly influenced by the earthquake’s intensity, their surroundings, and their evacuation training, which are all associated with the risk of injury. To protect victims and decrease the injury rate, educating individuals about earthquake rescue and evacuation exercises is of vital importance. This will hopefully both reduce victims’ extreme fear and prevent injury by fire or falling objects when panicking.

We also revealed that surviving a previous earthquake can protect victims from getting hurt. However, injury during the previous earthquake increased the injury rate in the current earthquake. Perhaps after the previous earthquake, victims were more willing to practice earthquake rescue training and drills, which may prevent future injuries. Victims who are injured again may be able to escape due to a physical disability or being “too afraid to move,” based on the psychological effects of experiencing another earthquake. Consequently, victims injured in previous earthquakes should receive mental health support directly after the earthquake. However, our results suggest that effective planning including earthquake evacuation knowledge and psychological counseling can save lives more efficiently. For example, in the Northridge, California earthquake, one of the most severe earthquakes to ever strike North America, 2 in 12 families had prepared themselves with some type of emergency evacuation plan and food. The same was true in Turkey, where families even fixed their overhead heavy material based on the experience of the previous earthquake where many things fell down. The government of earthquake-prone areas should conduct emergency evacuation exercises periodically, which is the most efficient way to protect people from disasters.

Being trapped was one of the key influencing factors for injury severity. Self- and mutual-aid plays an important role in the early time of rescue after earthquake. Being outside at the time of the earthquake or having escaped from the collapsing structure was crucial for survival. Many buildings were badly damaged after the Lushan earthquake. Almost half of the victims were trapped in the earthquake and most of them were buried under the ruins. Fortunately, 94% of the victims managed to be rescued in less than two hours. This could not have happened without the victims’ self-aid and mutual help in a timely fashion. In the research, 82% of victims got out of the ruin by themselves while only 1% got help from the professional rescue team. Additionally, 79% of them received medical first aid either by themselves or from their neighbors and relatives. Such a big percentage can really change the injury outcome and their loss. In the earthquakes in southern Italy and Mexico City, 80–90% of the victims assisted in post-earthquake rescue. In the Armenian earthquake, 89% of the survivors were rescued within 24 h [20]. Of the 130,000 persons who were injured, 14,000 were hospitalized. The self- and mutual-aid rate was much lower in the Armenian earthquake. Consequently, its death rate was higher than in the other earthquakes. The death and injury ratio was 0.19:1, while it was 0.01 in the Lushan earthquake found in this research. Such differences indicate how timely and efficient on-site medical care is critical to save victims’ lives. Self-aid training is rare for the people in the rural areas of west China. Basic medical training for the people living in earthquake-prone areas is strongly suggested to help them cope with such disasters more appropriately, which is the most cost-efficient way to save lives after terrible disasters. 

According to the result, a low percentage of victims, especially the severely injured or trapped ones, were rescued by the professional rescue team due to the delay in the rescue work. For these two kinds of victims, to save time is to save their lives and only the professional rescue team including the medical personnel can save them. Previous research has shown that deaths were 100 times higher and injury rates were more than five times higher among trapped than non-trapped victims [7,20]. However, it is often difficult for rescue forces to reach the earthquake area, especially directly after the earthquake when the traffic system is compromised. Consequently, most of the relief work was conducted within a few days by unprepared local people who concentrated on rescuing people sharing the same dwelling. The first 72 h after an earthquake are commonly recognized as the “gold time” for emergency rescue to save lives [21,22]. Consequently, rescue organizations and deployment of rescue teams is the key element in the earthquake emergency rescue. The number of helpers, their location, and deployment time should be organized according to the injury map in the earthquake area, which may reduce the casualty rate. Only in this way can we improve the efficiency and effect of the professional rescue team after the earthquake when there is a shortage of medical resources. 

## 5. Conclusions

This research focuses on the relationship between the impact of earthquake victims’ individual behavior response and earthquake-related injuries, while also considering the effectiveness of earthquake rescue measures. All data were collected through interviews with the earthquake victims. We used a questionnaire to get first-hand information about earthquake victims’ individual actions right after the earthquake. This study makes a significant contribution to the literature because research about this issue is severely lacking and there is an obvious connection between earthquake-related injury and individual behavioral characteristics. It is strongly suggested that victims obtain mental health support from medical practitioners and the government to minimize its negative effects. The initial reaction after an earthquake also played a vital role in victims’ trauma; therefore, earthquake-related experience and education may prevent injuries. Self-aid and mutual help played key roles in emergency, medical rescue efforts.

## Figures and Tables

**Table 1:** The demographic factors of Lushan earthquake victims after matching with geographic location factors.

		Without Injury (4875)		Injured (290)		Odds Ratios (OR)			
		Number (N)	%	N	%	OR	95% CI		*p*
Age *									
	15–35	1101	22.58	84	28.97	1.00			
	35–65	3377	69.27	153	52.76	0.86	0.61	1.22	0.396
	>65	397	8.14	53	18.28	2.01	1.27	3.18	0.003
**Sex**									
	Male	2396	49.15	137	47.24	1.00			
	Female	2479	50.85	153	52.76	1.01	0.79	1.30	0.914
**Education**									
	Above high school	813	16.68	77	26.55	1.00			
	Junior high school	2518	51.65	131	45.17	1.01	0.70	1.44	0.972
	Below primary school	1544	31.67	82	28.28	0.67	0.45	1.01	0.055
Occupation *									
	Farmer	4083	83.75	202	69.66	1.00			
	Teacher	73	1.50	20	6.90	3.33	1.81	6.13	0.000
	Government officer	48	0.98	1	0.34	0.42	0.06	3.14	0.398
	Worker	218	4.47	13	4.48	1.01	0.55	1.88	0.966
	Medical staff	32	0.66	10	3.45	4.35	1.88	10.08	0.001
	Student	242	4.96	24	8.28	1.75	0.99	3.09	0.056
	Business man	106	2.17	7	2.41	1.32	0.58	3.04	0.510
	Others	73	1.50	13	4.48	1.85	0.94	3.67	0.077
Marital Status *									
	Single	764	15.67	67	23.10	1.00			
	Married	4111	84.33	223	76.90	0.90	0.63	1.28	0.549

Newton-Raphson ridge method was used for parameter optimization and evaluation. The model is matched with geographic factors, the township location. The fitness of the model was evaluated by likelihood ratio test. * *p* < 0.05.

**Table 2:** Personal behavioral characteristics with injury situation.

		All	Not Injured	Injured	OR			
		N (%)	N (%)	N (%)	OR	95% CI		*p*
**Before the earthquake**								
Suffered from the Wenchuan earthquake or not *								
	Yes	4877 (94.42)	4621 (94.79)	256 (88.28)	1.00			
	No	288 (5.58)	254 (5.21)	34 (11.72)	2.11	1.41	3.17	0.000
Got injured during the earthquake or not *								
	Yes	195 (3.78)	167 (3.43)	28 (9.66)	1.00			
	No	4970 (96.22)	4708 (96.57)	262 (90.34)	0.34	0.22	0.53	<0.0001
Received earthquake evacuation training before the earthquake happened or not *								
	Yes	3153 (61.05)	3015 (61.85)	138 (47.59)	1.00			
	No	2012 (38.95)	1860 (38.15)	152 (52.41)	1.62	1.21	2.16	0.001
**During the earthquake**								
Fear level ^a^ (increase from 1 to 5) *								
	1	414 (8.02)	399 (8.18)	15 (5.17)	1.00			
	2	332 (6.43)	322 (6.61)	10 (3.45)	0.80	0.35	1.84	0.601
	3	598 (11.58)	579 (11.88)	19 (6.55)	0.87	0.43	1.76	0.703
	4	900 (17.42)	851 (17.46)	49 (16.9)	1.65	0.90	3.03	0.105
	5	2639 (51.09)	2454 (50.34)	185 (63.79)	1.93	1.11	3.35	0.020
	Forget	282 (5.46)	270 (5.54)	12 (4.14)	1.16	0.52	2.57	0.715
First reaction during the earthquake *								
	Maintain in the same place	1048 (20.29)	1001 (20.53)	47 (16.21)	1.00			
	Sit down	123 (2.38)	116 (2.38)	7 (2.41)	1.14	0.50	2.63	0.752
	Stand up	367 (7.11)	336 (6.89)	31 (10.69)	1.96	1.21	3.17	0.006
	Hidden under the desk or furniture	418 (8.09)	394 (8.08))	24 (8.28)	1.23	0.73	2.06	0.439
	Run out but trapped	329 (6.37)	267 (5.48)	62 (21.38)	4.19	2.76	6.36	<0.0001
	Run out of the building	2694 (52.16)	2576 (52.84)	118 (40.69)	0.95	0.66	1.35	0.754
	Others	186 (3.60)	185 (3.79)	1 (0.34)	0.11	0.02	0.82	0.031
**After earthquake**								
Trapped after the earthquake or not *								
	Yes	2073 (40.14	1869 (38.34)	204 (70.34)	2.50	1.87	3.34	<0.0001
	No	3092 (59.86)	3006 (61.66)	86 (29.66)	1.00			
Family members got trapped or not *								
	Yes	540 (10.45)	390 (8.00)	150 (51.72)	13.62	10.12	18.31	<0.0001
	No	4625 (89.55)	4485 (92.00)	140 (48.28)	1.00			

* The model is matched with geographic factors, the township location. The fitness of the model evaluated by likelihood ratio test. * *p* < 0.05. ^a^ Fear ranges from levels 1 to 5. Level 1 means not feeling terrified at all while Level 5 means feeling extremely terrified.
